# Protective Efficacy of Recombinant Influenza Hemagglutinin Ectodomain Fusions

**DOI:** 10.3390/v13091710

**Published:** 2021-08-27

**Authors:** Nidhi Mittal, Nayanika Sengupta, Sameer Kumar Malladi, Poorvi Reddy, Madhuraj Bhat, Raju S. Rajmani, Koen Sedeyn, Xavier Saelens, Somnath Dutta, Raghavan Varadarajan

**Affiliations:** 1 Molecular Biophysics Unit (MBU), Indian Institute of Science, Bengaluru 560012, India; nidhimittal@iisc.ac.in (N.M.); nayanikas@iisc.ac.in (N.S.); sameerm@iisc.ac.in (S.K.M.); rsrajmani@iisc.ac.in (R.S.R.); somnath@iisc.ac.in (S.D.); 2 Mynvax Private Limited, ES12, Entrepreneurship Centre, SID, Indian Institute of Science, Bengaluru 560012, India; poorvi.reddy@mynvax.com (P.R.); madhuraj.bhat@mynvax.com (M.B.); 3 VIB-UGent Center for Medical Biotechnology, VIB, 9052 Ghent, Belgium; koen.sedeyn@vib-ugent.be (K.S.); xavier.saelens@vib-ugent.be (X.S.); 4 Department of Biochemistry and Microbiology, Ghent University, 9052 Ghent, Belgium

**Keywords:** influenza virus, hemagglutinin, immunogen, mouse immunization, neutralization, linker, trimerization, mouse-adapted

## Abstract

In current seasonal influenza vaccines, neutralizing antibody titers directed against the hemagglutinin surface protein are the primary correlate of protection. These vaccines are, therefore, quantitated in terms of their hemagglutinin content. Adding other influenza surface proteins, such as neuraminidase and M2e, to current quadrivalent influenza vaccines would likely enhance vaccine efficacy. However, this would come with increased manufacturing complexity and cost. To address this issue, as a proof of principle, we have designed genetic fusions of hemagglutinin ectodomains from H3 and H1 influenza A subtypes. These recombinant H1-H3 hemagglutinin ectodomain fusions could be transiently expressed at high yield in mammalian cell culture using Expi293F suspension cells. Fusions were trimeric, and as stable in solution as their individual trimeric counterparts. Furthermore, the H1-H3 fusion constructs were antigenically intact based on their reactivity with a set of conformation-specific monoclonal antibodies. H1-H3 hemagglutinin ectodomain fusion immunogens, when formulated with the MF59 equivalent adjuvant squalene-in-water emulsion (SWE), induced H1 and H3-specific humoral immune responses equivalent to those induced with an equimolar mixture of individually expressed H1 and H3 ectodomains. Mice immunized with these ectodomain fusions were protected against challenge with heterologous H1N1 (Bel/09) and H3N2 (X-31) mouse-adapted viruses with higher neutralizing antibody titers against the H1N1 virus. Use of such ectodomain-fused immunogens would reduce the number of components in a vaccine formulation and allow for the inclusion of other protective antigens to increase influenza vaccine efficacy.

## 1. Introduction

Influenza is a major global pathogen that causes significant morbidity and 290,000–650,000 human deaths annually, with a potential 10–100-fold higher toll in a pandemic [1]. Influenza viruses are enveloped, negative-sense, segmented, single-stranded RNA viruses of the *Orthomyxoviridae* family [2]. Based on antigenic differences, influenza viruses are categorized into four genera: A, B, C, and D. Influenza A viruses are broadly classified into two phylogenetic groups based on hemagglutinin (HA) subtypes: group 1 viruses comprise H1, H2, H5, H6, H8, H9, H11, H12, H13, H16, H17, and H18, and group 2 viruses include H3, H4, H7, H10, H14, and H15 [3]. Influenza B viruses are categorized into Yamagata and Victoria phylogenetic lineages [4]. Currently H1N1 and H3N2 strains of influenza A and Victoria and Yamagata lineages of influenza B viruses co-circulate in the human population and cause seasonal epidemics.

Influenza vaccination is the preferred and most cost-effective intervention tool currently available to prevent influenza virus infection and disease. Licensed influenza vaccines include inactivated whole or split viruses, (recombinant) viral subunit, and live attenuated vaccines [5]. Seasonal influenza vaccines contain viral strains or hemagglutinins (HAs) closely related to the putative upcoming seasonal strains of influenza A viruses and influenza B viruses. Most current vaccine approaches almost exclusively focus on raising a humoral immune response against hemagglutinin (HA)—the immunodominant, surface glycoprotein of influenza virus essential for viral entry and fusion with the host cell membrane [6,7]. Hemagglutinin is synthesized as a precursor polypeptide (HA_0_) chain that associates non-covalently and folds to form homotrimers [8]. Each hemagglutinin monomer comprises two structurally distinct regions—a membrane distal, globular head domain, consisting predominantly of the HA1 subunit, and a membrane-proximal, helical, stem domain composed mainly of the HA2 subunit [7]. Hemagglutinin-specific antibodies elicited during infection or vaccination are often neutralizing [3,9]. Neutralizing antibodies (nAb) predominantly target epitopes located in the immunodominant, globular head domain, while a subset of neutralizing antibodies recognize and bind to more conserved epitopes in the stem domain of hemagglutinin [3]. Both HA head and stem-directed antibodies have been shown as independent immune correlates of protection against influenza infection in humans [10]. Due to the continuous antigenic drift of seasonal influenza viruses and the escape of drift variants from pre-existing immunity elicited by previous infections or vaccination, seasonal vaccines have an average efficacy of ~50% [11,12,13]. However, this can be considerably lower when the vaccine strain mismatches currently circulating strains, and current vaccines are ineffective against pandemic influenza viruses.

Furthermore, vaccine strains need to be updated every year [14]. Thus, to improve immunogenicity and provide broad-range, long-lasting protection, conserved antigens of influenza viruses, such as the hemagglutinin stem, neuraminidase, matrix, and internal proteins, have been explored to develop a ‘universal influenza vaccine’ [15]. Antibodies against neuraminidase (NA), the other major surface glycoprotein which mediates viral egress, are known to protect against influenza virus infection, and neuraminidase inhibition (NAI) titers have been identified as a correlate of protection [16,17]. Recently, neutralizing anti-NA antibodies against influenza A and B viruses have been identified, suggesting, correctly folded and immunologically relevant NA antigens can induce broadly protective antibody responses [18,19]. In addition, the N-terminal extracellular domain of matrix protein 2 (M2e) reduces viral replication in infected cells and confers cross-protection against different strains of influenza viruses [15,20,21,22]. Vaccines derived from egg grown, inactivated virus constitute the bulk of current influenza virus vaccines. While these might be expected to include all the above immunogens, in practice the bulk of the neutralizing response elicited by these vaccines is detected against the globular head of HA and these vaccines contain variable amounts of NA. Recently, a quadrivalent recombinant seasonal vaccine, consisting of insect cell expressed HA from H1, H3 and the two B lineages has been developed and widely deployed [23,24].

One of the primary drawbacks of including other antigenic components in the vaccine formulations is the technical difficulty of adding additional elements to an already quadrivalent HA vaccine formulation. Genetic fusions of HA ectodomains from influenza A virus subtypes are a potential approach to overcome this issue. However, it was unclear if this could be done without severely compromising antigen conformational integrity and yield. To study these issues, we designed recombinant hemagglutinin ectofusion constructs, where an H3 hemagglutinin ectodomain was genetically fused with an H1 ectodomain with the help of a flexible linker and heterologous trimerization sequences. We show that the resulting designed, recombinantly expressed, H1–H3 HA ectodomain-fused (hereafter abbreviated to ectofusion) immunogens elicit both H1 and H3 specific humoral immune responses in mice and protect against heterologous challenge with both H1 and H3 influenza viruses, comparable to an equimolar mixture of separately expressed and purified individual H1 and H3 ectodomains. These recombinant HA ectofusion based immunogens can potentially help minimize the total number of HA constructs that need to be produced under Good Manufacturing Practices (GMP), allowing for additional non-HA components to be included, thus boosting vaccine efficacy. Relative to conventional inactivated virus, use of recombinant protein-based immunogens allows for improved characterization of individual components, adjustment of their relative stoichiometry, avoids both the presence of egg-adapted mutations and the requirement for large numbers of pathogen free eggs, and allows for increased scalability and rapidity of manufacture.

## 2. Materials and Methods

### 2.1. Cell Line, Antibodies and Viruses

The Expi293 transient expression system was used for rapid and high-yield production of designed immunogens and antibodies from mammalian cells. Expi293 suspension-adapted cells, derived from the human 293F cell line, were maintained in Expi293™ Expression Media (Catalog Number: A14635, Gibco, ThermoFisher). In our hands, these gave considerably higher (~3–10 fold) yields than adherent 293 or suspension 293F cells. Use of mammalian cells enabled testing of multiple constructs in a short period of time and also ensured native glycosylation. Light and heavy chain genes of the CR9114, C05 and MA2077 antibodies were synthesized and cloned into the pCDNA3.4 by GenScript [25,26,27]. Antibodies were transiently expressed in Expi293F cells and were purified by protein A affinity chromatography. Purified CR9114 (stem-directed), C05 (H3 head-directed), and MA2077 (H1 head-directed) antibodies were used in surface plasmon resonance (SPR) binding studies to evaluate the conformational integrity of designed ectofusion immunogens. The infectious influenza viruses A/Guangdong-Maonan/SWL1536/2019 (NIBSC code: 19/294) and A/HongKong/2671/2019 (NIBSC code: 19/292) were obtained from the NIBSC, UK.

### 2.2. Cloning of H1H3 HA Ectofusion Constructs

The amino acid sequences of H1, H3 hemagglutinin ectodomains were derived from A/Hawaii/70/2019 (GenBank Protein Accession: QGW43678.1) and A/Hong Kong/45/2019 (GISAID Accession: EPI1691930) influenza strains, respectively. Genes for H1, H3 ectodomains (mMH1_02TE and mMH3_02TE, respectively) were human codon-optimized and cloned under control of the cytomegalovirus (CMV) promoter by GenScript Inc. (Piscataway, NJ, USA). The HA ectofusion construct genes (mMH3H1F_02TE and mMH3FH1F_02TE) were cloned under control of the CMV promoter using three fragment Gibson recombination [28]. The coding region of the individual H1 and H3 ectodomain expression constructs consisted of a tissue plasminogen activator (tPA) signal sequence followed by the HA derivative linked to a cleavable foldon trimerization domain and histidine tag [29]. tPA signal sequence at the N-terminus of proteins facilitated protein secretion in culture supernatant. The 10X His- tag at the C-terminus of proteins enabled purification of proteins.

### 2.3. Protein Expression and Purification

Polyhistidine-tagged recombinant proteins mMH1_02TE, mMH3_02TE, mMH3H1F_02TE, and mMH3FH1F_02TE were individually transiently transfected and expressed, extracellularly, using Expi293F cells as described previously [30]. Briefly, plasmid DNA (1 µg/mL) and ExpiFectamine™ 293 reagent (Gibco, ThermoFisher Scientific, Waltham, NY, USA) were diluted with Opti-MEM™ I reduced serum media (Gibco, ThermoFisher Scientific) and were incubated for 5 min at room temperature. For plasmid DNA complexation, a diluted ExpiFectamine™ 293 reagent was mixed with diluted plasmid DNA. It was incubated at room temperature for 20 min, and the solutions were then slowly added to Expi293F cells (3 × 10^6^ cells/mL). Cells were then incubated in a 37 °C incubator with a humified atmosphere of 8% CO_2_ on an orbital shaker. Post 18–22 h of transfection, ExpiFectamine™ 293 Transfection Enhancer 1 and Enhancer 2 (Gibco, ThermoFisher Scientific) were added. Culture was harvested five days post transfection, and proteins were purified from culture supernatant by nickel affinity chromatography. The two-fold diluted supernatant was incubated with phosphate buffer saline (PBS) (pH 7.4) equilibrated Ni-Sepharose 6 Fast Flow resin (GE Healthcare) for 4–5 h at 4 °C under mild-mixing conditions to facilitate binding. Unbound and non-specific proteins were removed by passing ten column volumes of wash buffer (PBS + 25 mM Imidazole, pH 7.4). Bound proteins were eluted from the column using an imidazole gradient (50–500 mM imidazole in PBS buffer, pH 7.4). Eluted fractions containing the protein of interest were pooled and dialyzed against PBS (pH 7.4) using a 6–8 kDa (MWCO) dialysis membrane (Spectrum Labs). Protein purity was analyzed on SDS-PAGE followed by Coomassie staining.

### 2.4. Differential Scanning Fluorimetry

The thermal stability of the HA ectodomains and ectofusion immunogens were determined using nano-DSF (differential scanning fluorimetry) on a Prometheus NT.48 instrument (Nano Temper) [31]. Thermal unfolding of protein samples at a concentration of 5 μM was monitored at a rate of 1 °C/min in a range from 20 °C to 90 °C. The normalized first derivative of fluorescence ratio (350 nm/330 nm) was plotted against temperature using Prism v8.4.3 (Graph Pad Software, San Diego, CA, USA).

### 2.5. Sample Preparation and Data Collection for Negative Staining Transmission Electron Microscopy (NS-TEM)

Purified HA ectofusion complexes (mMH3H1F_02TE, mMH3FH1F_02TE) were analyzed for the nature of particle distribution and overall homogeneity using NS-TEM. Carbon coated copper TEM grids were glow discharged in a GloQube glow discharge system for 30 s prior to sample addition. Purified samples (3.5 μL of 0.1 mg/mL) were applied onto the Cu grids and incubated at room temperature for 1 min. Excess sample was carefully blotted off with a Whatman filter paper. This was followed by negative staining using a 1% solution of freshly prepared uranyl acetate. Data were acquired on a 120 kV Talos L120C room temperature electron microscope, equipped with a bottom mounted Ceta camera (4k × 4k) at a calibrated pixel size of 2.42 Å/pixel at specimen level.

### 2.6. Negative Staining TEM Data Processing

Two sets of raw micrographs for mMH3H1F_02TE and mMH3FH1F_02TE were imported into EMAN 2.2 for further assessment of the protein complexes [32]. Approximately 2076 mMH3H1F_02TE particles and 2378 mMH3FH1F_02TE particles were manually picked and extracted using e2boxer.py in EMAN2.2 software. Reference-free 2D classifications of the particle projections were calculated using simple_prime2D of SIMPLE 2.0 software [33].

### 2.7. Size Exclusion Chromatography-Multi-Angle Light Scattering (SEC-MALS)

Size exclusion chromatography was performed using a Superdex-200 10/300 analytical gel filtration column (GE Healthcare). Purified protein samples (75 μg) were injected into the column, equilibrated with 1X PBS buffer (pH 7.4), and eluted at room temperature at a 0.5 mL/min flow rate. In-line UV detector (Shimadzu Corporation, Kyoto, Japan), refractive index detector (WATERS 2414), and triple angle MALS scattering (miniDAWN TREOS, Wyatt Technology Corporation, Santa Barbara, CA, USA) detectors were used for molar mass determination (dn/dc = 0.185 mL mg^−1^). ASTRA™ software (Wyatt Technology) was used for data analysis. The theoretical molecular weight of proteins was calculated using the ExPASy-ProtParam tool. An addition of 1.5 kDa per glycosylation site was added to the overall molecular weight.

### 2.8. Binding Affinity Measurement Using SPR

The binding affinity of the HA ectofusion immunogens to conformation-specific antibodies was measured using SPR (ProteOn XPR36 Protein Interaction Array V.3.1, Bio-Rad). First, the GLM sensor chip was activated using EDC and sulfo-NHS (Sigma, St. Louis, MO, USA), followed by Protein G (10 μg/mL) (Sigma) immobilization in various channels for 300 s (30 μL/min) in the presence of 10 mM sodium acetate buffer (pH 4.0) and then using 1 M ethanolamine excess sulfo-NHS esters quenched. Nearly 1000 response units (RU) of monoclonal antibodies MAb2077, C05, and CR9114 were immobilized at a flow rate of 5 μg/mL for 100 s. Five different concentrations (100 nM, 50 nM, 25 nM, 12.5 nM, and 6.25 nM) of HA ectofusion immunogens were passed at a flow rate of 30 μL/min for 100 s over the chip surface, followed by a dissociation step of 200 s. After each kinetic assay, the chip was regenerated in 0.1 M Glycine-HCl (pH 2.7), and kinetic parameters were obtained by fitting the data to a simple 1:1 Langmuir interaction model using Proteon Manager.

### 2.9. Mice Immunization and Challenge Studies

All immunization experiments were carried out at the Central Animal Facility, Indian Institute of Science. Groups of 5 BALB/c mice (6–8 weeks old) were immunized intramuscularly with 20 μg of test ectofusion immunogen along with SWE adjuvant (1:1 *v/v* antigen: SWE ratio per animal per dose, i.e., 20 μg of antigen in 50 μL of PBS and 50 μL of SWE) at day 0 (prime) and day 21 (boost). Squalene-in water emulsion (SWE) adjuvant composition is similar to MF59 adjuvant and is free of intellectual property rights facilitating its open access for use. Moreover, it was shown to be safe and effective in preclinical studies for various vaccine candidates [34]. Adjuvant-treated mice and mice immunized with 20 μg of H1 and 20 μg of H3 HA ectodomains, mixed in an equimolar ratio with SWE adjuvant, were used as controls. Sera samples were isolated from the bleeds drawn before prime (day -1), post-prime (day 14), and post-boost (day 35). Twenty-one days after secondary immunization, mice were anesthetized and were intranasally challenged with 10 MLD_50_ mouse-adapted Bel/09 (H1N1) or X-31 (H3N2) virus in 20μL of PBS. X-31 (H3N2) is engineered to express surface HA glycoprotein genes of A/Aichi/2/68 (H3N2) and the remaining genes are derived from A/Puerto Rico/8/34 virus [35]. Survival and weight loss of the challenged and control mice groups were monitored daily for 14 days post-challenge. The weight of individual mice (surviving) was recorded at each time point. The weight change differences amongst adjuvant-treated and mice immunized with immunogens were analyzed by performing multiple Student’s *t*-test with Bonferroni Dunn’s correction method.

### 2.10. Determination of Serum Antibody Titers

Enzyme linked immunosorbent assay (ELISA) was performed to determine serum antibody titers against test immunogens. Briefly, 4 μg/mL of test immunogens (50 μL/well) were coated on 96 well Nunc plates (Thermo Fisher Scientific, Rochester, NY, USA) for 2 h at room temperature, under constant shaking at 300 rpm on a MixMate thermomixer (Eppendorf AG, Hamburg, Germany). After washing with 1X PBS containing 0.05% Tween 20 (PBST) four times, plates were blocked with 3% skimmed milk in PBST (blocking buffer) for 1 h at room temperature. Antisera raised against test immunogens were serially diluted four-fold in blocking buffer and were added to wells. Plates were then incubated for 1 h, 300 rpm at room temperature, followed by three washes with PBST, after which 50 μL of ALP-conjugated goat anti-mouse IgG secondary antibody (1:5000) diluted in blocking buffer was added (50 μL/well) and incubated at room temperature for 1 h. Plates were washed with PBST (four times) followed by the addition of pNPP liquid (SIGMA-ALDRICH) substrate (50 μL) to each well and then incubated at 37 °C for 30 min. Optical density at 405 nm was measured. The highest serum dilution possessing signal above 0.2 O. D at 405 nm was considered the endpoint titer for ELISA. Data were plotted using Prism v8.4.3 (Graph Pad Software, San Diego, CA, USA). Two-tailed Student’s *t*-test was performed for pairwise ELISA endpoint titer comparisons.

### 2.11. Hemagglutinin Inhibition (HI) Assay

HI titers to vaccine-matched and challenge-matched viruses were tested with mice sera. Immunized mice sera were heat-inactivated and treated with receptor destroying enzyme (RDE, SIGMA-ALDRICH) before use. Sera were two-fold serially diluted with cold PBS buffer and incubated with the indicated viruses (4 HAU/well), and then incubated with 1% Guinea pig red blood cells (RBC) for 30 min at room temperature. Hemagglutinin inhibition (HI) titers were recorded as the highest serum dilution at which no agglutination was observed. Two-tailed Student’s *t*-test was performed for pairwise HI titer comparisons.

### 2.12. Microneutralization Assay

Viruses were grown in Madin-Darby Canine Kidney (MDCK) cells in the presence of TPCK-treated trypsin (1 µg/mL) and stored at −70 °C. Immune mice sera samples were heat-inactivated and treated with receptor destroying enzyme (RDE, SIGMA-ALDRICH ch) before use. Immunized sera samples were two-fold serially diluted and incubated for 1 h at 37 °C in 5% CO_2_ with 50 TCID_50_ viruses. Serum-virus mixture was then transferred to 96 well plates, and 1.5 × 10^5^ MDCK-London cells/mL were added to each well. Plates were then incubated for 48 h at 37 °C in 5% CO_2,_ and cytopathic effects were observed. MN assay for matched virus was only performed with group I, III, V (H1) and group II, IV and VI (H3) as groups (I and II), (III and IV), (V and VI) were identical in terms of their immunogens, only the challenge virus was different. The neutralization titer in the assay is the highest serum dilution at which no cytopathic effect was observed. Two-tailed Student’s *t*-test was performed for pairwise MN titer comparisons.

## 3. Results

### 3.1. H1H3 Ectofusion Immunogen Design

**Figure 1:**
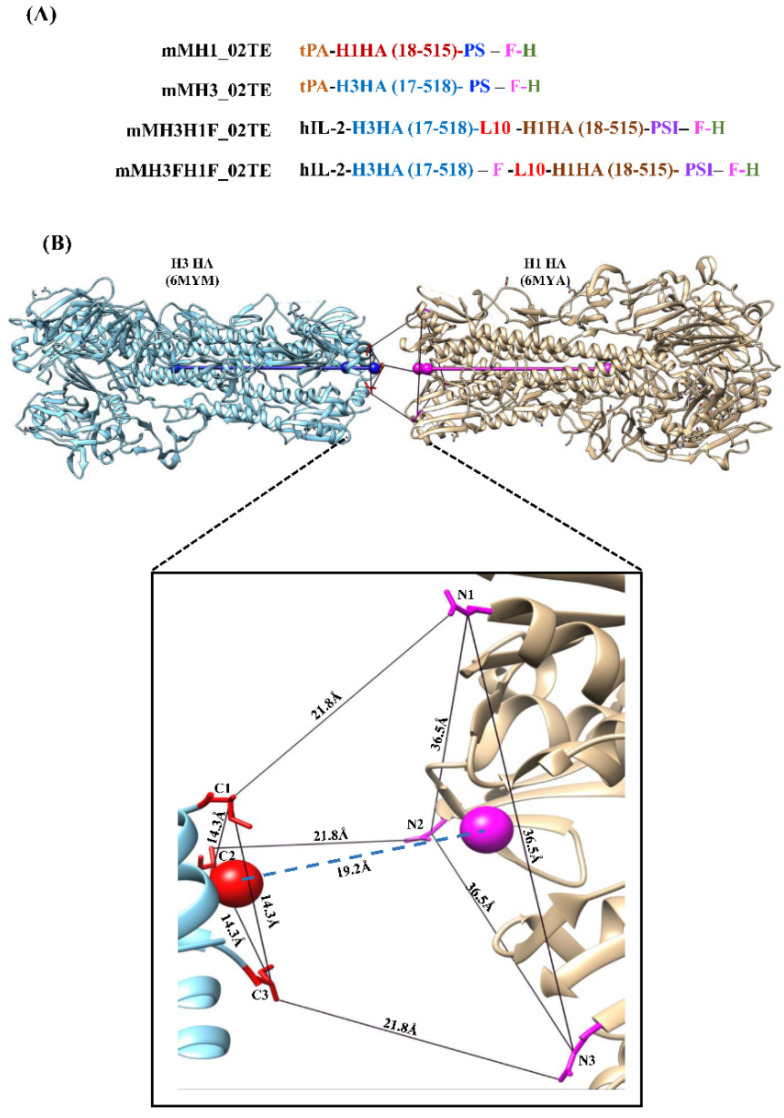
Hemagglutinin Ectofusion Immunogen Design. (**A**) Schematic representation of hemagglutinin (HA) immunogen sequences. L10, PS, PSI, F, H represent a ten-residue linker, HRV3C protease cleavage site, TEV cleavage site, foldon and histidine tag respectively. tPA and hIL-2 are signal peptides. H1 and H3 ectodomains consist of N-terminal tPA signal sequence followed by the HA ectodomain linked to a cleavable foldon trimerization domain and a C-terminal histidine tag. mMH3H1F_02TE immunogen comprises of hIL-2 signal sequence followed by the H3 HA ectodomain, which is connected with the H1 HA ectodomain by a flexible 10 residue linker followed by a cleavable foldon trimerization domain and a C-terminal histidine tag. mMH3FH1F_02TE is similar to the above fusion but contains an additional foldon sequence between the H3 and H1 ectodomains. (**B**) For hemagglutinin ectofusion immunogen design, H3 ectodomain (PDB: 6YM) and H1 ectodomain (PDB: 6MYA) were aligned along their 3-fold symmetry axes, shown in blue and pink, respectively, using UCSF Chimera Visualization software. Triangular planes defined by the three N and three C-termini respectively were separated by a perpendicular distance of 19.2 Å, to avoid steric clashes between molecules. In the hemagglutinin ectofusion modeled structure, the C-termini of H3 and N-termini of H1 HA are minimally 21.8 Å apart and were, therefore, connected using a ten-residue long flexible ‘GSA’ linker.

Hemagglutinin ectodomains from influenza A subtypes H1 and H3 were genetically fused. Two ectofusion immunogens were designed, containing either a single foldon trimerization motif at the C- terminus of the H1 hemagglutinin ectodomain (designated as mMH3H1F_02TE), or two foldons, one foldon at the C-terminus of the H3 hemagglutinin ectodomain and the other at the C-terminus of the H1 hemagglutinin ectodomain (designated as mMH3FH1F_02TE) (Figure 1A).

In the mMH3H1F_02TE ectofusion, the C-terminus of the H3 HA ectodomain (residues 17-518) was connected to the N-terminus of the H1 HA ectodomain (18-515) with a soluble GSA linker. The length of the GSA linker for connecting H3 to the H1 hemagglutinin ectodomain was calculated using UCSF Chimera Visualization software. The three C-termini of H3 HA are situated at the vertices of an equilateral triangle with an edge of length 14.3Å. The corresponding distance between N- termini of H1 HA is 36.5 Å. The H3 and H1 HA ectodomains were aligned along their three-fold symmetry axes. To avoid a steric clash between the H1 and H3 hemagglutinins, planes defined by the three N and three C-termini, respectively, were separated by a perpendicular distance of 19.2Å. The resultant shortest distance was determined between the C-termini of H3 and the N-termini of H1 HA to be 21.8Å. A ten-residue flexible GSA linker (L10) accommodates this distance, enabling the genetic fusion of the H3 ectodomain with the H1 ectodomain (Figure 1B). Similarly, the C-terminus of foldon was linked to the N-terminus of H1 in the mMH3FH1F_02TE construct.

### 3.2. Biophysical Characterization of H1H3 HA Ectofusion Immunogens

**Figure 2:**
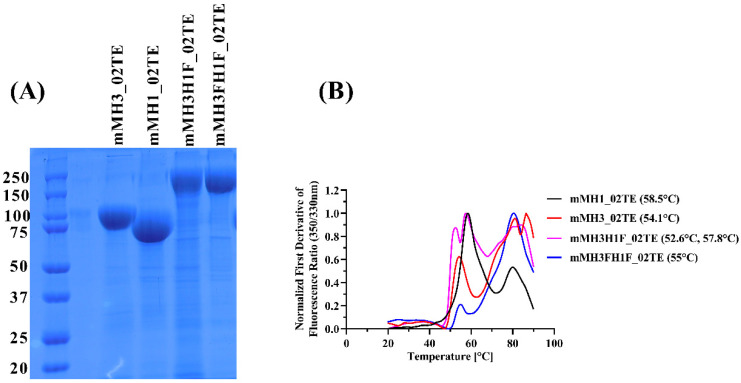
Expression and characterization of hemagglutinin immunogens. (**A**) SDS-PAGE profile of nickel affinity purified HA immunogens, expressed in Expi293F cell culture. Molecular weights of marker proteins in kDa are shown. The purified H3 and H1 ectodomains show apparent molecular weights of ~85 kDa and ~75 kDa, respectively. The hemagglutinin ectofusion immunogens (mMH3H1F_02TE and mMH3H1F_02TE) have an apparent molecular weight of ~150 kDa. (**B**) Equilibrium thermal unfolding measured using nano-DSF. The normalized first derivative of fluorescence intensity ratio (350 nm/330 nm) is plotted as a function of temperature (°C). The high-temperature peak at ~80 °C is likely due to protein aggregation.

The designed hemagglutinin ectofusion immunogens, and individual H1, H3 HA ectodomains were transiently expressed in Expi293F cells and purified from Expi293F culture supernatant using single-step nickel affinity chromatography. Coomassie-stained non-reducing SDS-PAGE was used to assess the purity of fusion construct designs, which was approximately 90–95% (Figure 2A). The yield of mMH3H1F_02TE and mMH3FH1F_02TE ectofusion immunogens were ~3.5 mg/mL and ~9 mg/mL, respectively. In contrast to ectofusion immunogens, the individual H1, H3 ectodomains (mMH1_02TE and mMH3_02TE) have better yields of ~24 mg/mL and 25 mg/mL, respectively. Nano-DSF data revealed that ectofusion immunogens mMH3H1F_02TE and mMH3FH1F_02TE are thermostable and have similar equilibrium thermal unfolding profiles as the individual H1 (mMH1_02TE) and H3 (mMH3_02TE) ectodomains (Figure 2B). mMH3H1F_02TE has a slightly higher apparent melting temperature (T_m_~57.3 °C) than mMH3FH1F_02TE (T_m_~54.4 °C).

**Figure 3:**
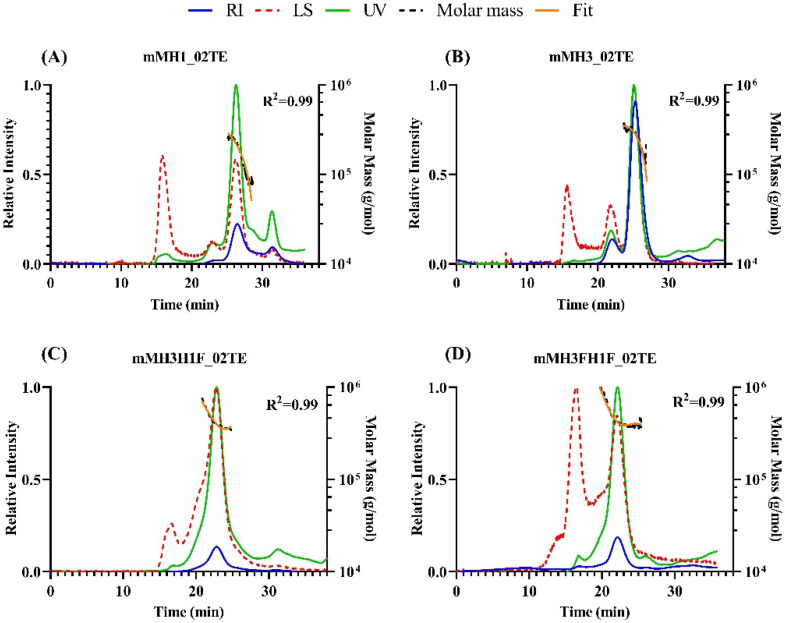
SEC-MALS profile of purified hemagglutinin immunogens. Relative intensities of all traces [UV (green), refractive index (blue), and light scattering (red)] are plotted as a function of elution time. Orange trace and black trace representing the fit and molar mass for trimeric fractions of HA immunogens are shown. (**A**) mMH1_02TE, (**B**) mMH3_02TE, (**C**) mMH3H1F_02TE, (**D**) mMH3FH1F_02TE.

The oligomeric state of the designed HA ectofusion immunogens and individual HA ectodomains was probed by size exclusion chromatography—multi-angle light scattering experiments (SEC-MALS). mMH1_02TE, mMH3_02TE, mMH3H1F_02TE and mMH3FH1F_02TE exist as trimers in solution as the calculated molecular weights (mMH1_02TE: 214.5 (±0.4%) kDa; mMH3_02TE: 284.1 (±0.4%) kDa; mMH3H1F_02TE: 402.1 (±0.7%) kDa; and mMH3FH1F_02TE: 426.0 (±0.2%) kDa) were in good agreement with the expected theoretical molecular weight for the corresponding trimers (mMH1_02TE: 231 kDa; mMH3_02TE: 252 kDa; mMH3H1F_02TE: 432 kDa; and mMH3FH1F_02TE: 441 kDa) (Figure 3).

**Figure 4:**
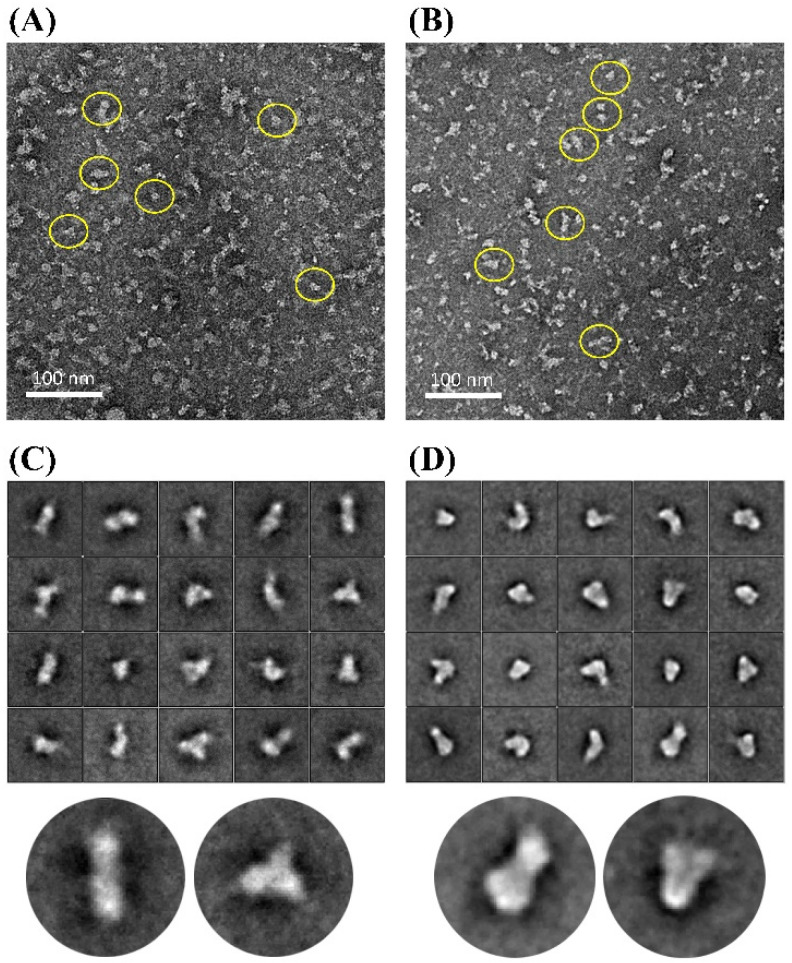
Negative staining transmission electron microscopic analysis of HA ectofusion immunogens (**A**) Negative staining raw micrograph of mMH3H1F_02TE ectofusion showing elongated conjugates. (**B**) Negative staining raw micrograph of mMH3FH1F_02TE ectofusion showing elongated conjugates. Particles have been marked within the yellow boundary. (**C**) Reference-free 2D class averages of mMH3H1F_02TE ectofusion showing the various orientations of the immunogens. Lower panel denotes magnified view of two representative classes of the molecule. (**D**) Reference-free 2D class averages of mMH3FH1F_02TE ectofusion showing the various orientations of the immunogens. Lower panel denotes magnified view of two representative classes of the molecule.

Further characterization of the HA ectofusion immunogens was performed using room temperature negative staining transmission electron microscopy (NS-TEM). TEM revealed monodispersed immunogen particles in several orientations (Figure 4A,B). The 2D class averages of mMH3H1F_02TE were predominantly tubular with trimeric symmetry (Figure 4C). In contrast, mMH3FH1F_02TE construct appeared to adopt a more compact packing of the two ectodomains while also existing as trimeric molecules (Figure 4D).

### 3.3. H1H3 Ectofusion Immunogens Bind Conformation-Specific Broadly Neutralizing Antibodies (bnAbs)

**Figure 5:**
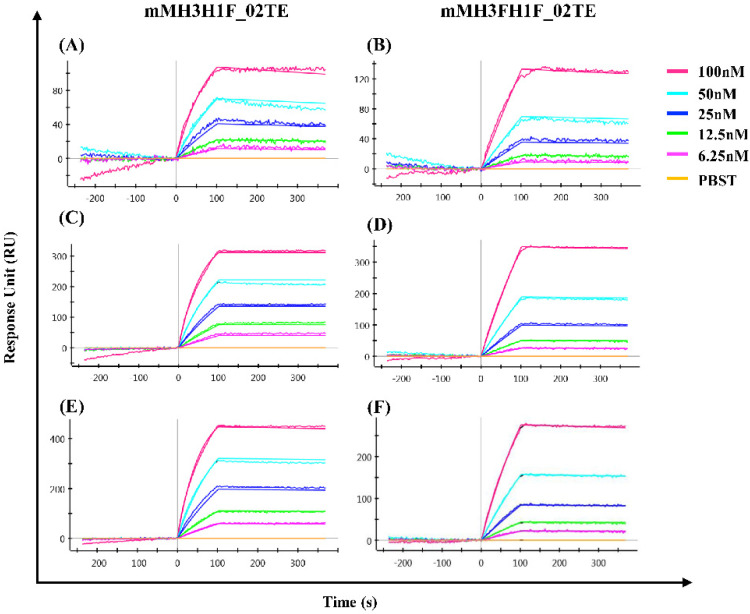
Hemagglutinin ectofusion immunogens bind conformation-specific broadly neutralizing antibodies. The overlays of binding kinetics monitored by SPR at different concentrations of HA ectofusion immunogens to different conformation-specific antibodies (**A**,**B**) MA 2077, (**C**,**D**) C05, and (**E**,**F**) CR9114 are shown. MA2077 and C05 are H1 and H3 specific HA head-directed mAbs respectively, while CR9114 is a pan-influenza specific, HA stem-directed antibody.

Surface plasmon resonance was performed to assess the antigenicity and proper folding of designed hemagglutinin ectofusion immunogens. The binding of the designed H1H3 HA ectofusion immunogens to a panel of bnAbs (CR9114, C05, MA2077) was determined. CR9114 is a HA stem-directed broadly neutralizing antibody (bnAb) while C05 is an H3 head-directed bnAb [25,27]. MA2077 is an H1 specific, head-directed neutralizing antibody [26]. Ectofusion constructs show binding with MA2077, C05, and CR9114 (Figure 5, Table 1). The ability of ectofusion constructs to bind these bnAbs with high affinity and low off rates provides strong validation of their proper folding and antigenicity.

### 3.4. H1H3 HA Ectofusion Immunogens Elicit a Protective Immune Response in Mice

**Figure 6:**
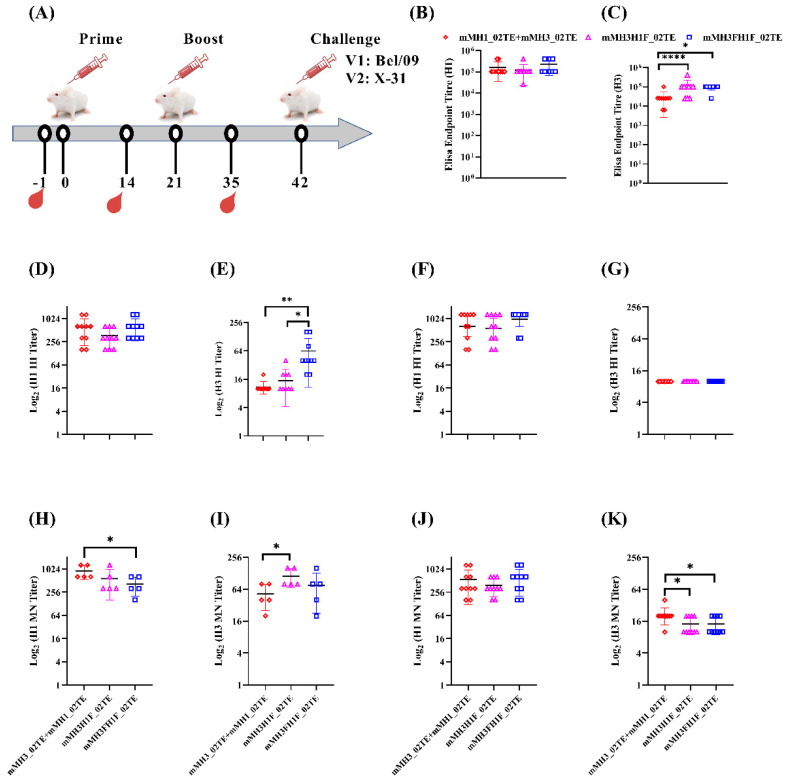
Immunogenicity of hemagglutinin ectofusion immunogens in mice. (**A**) Schematic representation of immunization schedule. Serum titers were obtained after two immunizations with SWE formulated immunogens. (**B**,**C**) ELISA endpoint titers against tagless H1 and H3 hemagglutinin ectodomains, respectively. (**D**,**E**) HI titers against homologous A/Guangdong−Maonan/SWL1536/2019 (H1N1) and A/Hongkong/2671/2019 (H3N2) viruses, respectively. (**F**,**G**) HI titers against heterologous Bel/09 and X−31 viruses, respectively. (**H**,**I**) MN titers against homologous A/Guangdong−Maonan/SWL1536/2019 (H1N1) and A/Hongkong/2671/2019 (H3N2) viruses, respectively. (**J**,**K**) MN titers against heterologous Bel/09 and X−31 viruses, respectively. Two−tailed Student’s *t*−test was performed for pairwise ELISA endpoint titer, HI titer and MN titer comparisons (* indicates *p* < 0.05, ** indicates *p* < 0.01, and **** indicates *p* < 0.0001).

We further evaluated the immunogenicity of the hemagglutinin ectofusion immunogens: mMH3H1F_02TE, mMH3FH1F_02TE relative to the individual H1 and H3 ectodomains mixed in equimolar amounts (mMH1_02TE + mMH3_02TE) in mice. Animals were intramuscularly immunized with 20 µg of SWE adjuvanted immunogen in a prime-boost regimen with a three-week interval. Adjuvant-treated mice were used as controls. H1 and H3 hemagglutinin-specific antibodies in sera samples were measured 14 days post-boost using ELISA, HI, and MN assays. Interestingly, ectofusion immunogens elicited both H1 and H3 specific humoral immune responses, comparable to individual H1 and H3 hemagglutinin ectodomains mixed in an equimolar ratio (mMH1_02TE + mMH3_02TE). The ELISA endpoint titers range from 25,600–409,600 against untagged H1 and H3 proteins. H1 HI titers against both homologous (A/Guangdong-Maonan/SWL1536/2019) and heterologous (A/Belgium/145-MA/2009 (Bel/09)) viruses were high (160–1280) for all three groups. However, H3 HI titers were low in all cases. Similarly, microneutralization titers were high against H1 virus (160–1280) and low for H3 virus (10–40) for all groups for both homologous and heterologous virus (Figure 6, Table S1). Since there were two identical groups for each immunogen which differed only in the challenge virus, microneutralization assays with homologous virus were only carried with one of the two groups (Figure 6H,I).

**Figure 7:**
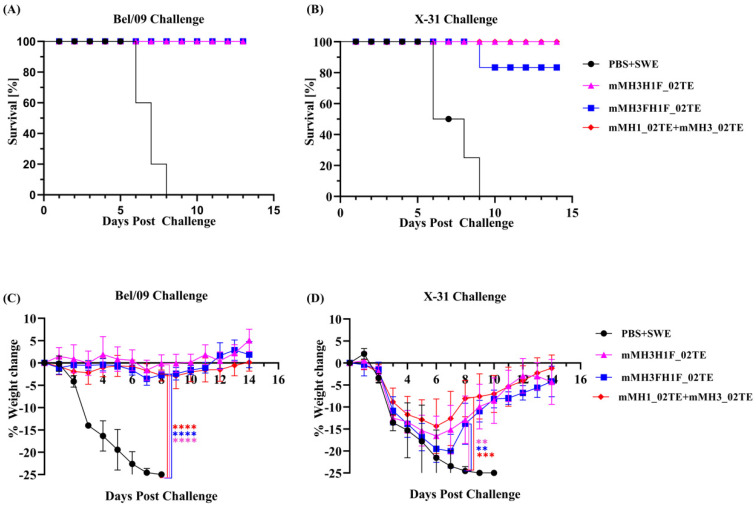
Hemagglutinin ectofusion immunogens protect mice against challenge. Mice (*n* = 5/group) were primed (day 0) and boosted (day 21) with 20 µg of the indicated immunogens and challenged intranasally 21 days after the boost with 10 MLD_50_ of mouse-adapted Bel/09 and X−31 viruses. (**A**,**B**) survival and (**C**,**D**) percentage weight change was monitored for 14 days post−challenge. Adjuvant-treated mice were used as controls. In contrast to unimmunized mice controls, mMH3H1F_02TE and mMH1FH3F_02TE conferred protection, similar to that of the mMH3_02TE + mMH1_02TE mixture. Multiple Student’s *t*−test was performed with Bonferroni Dunn’s correction method for pairwise weight change comparisons (** indicates *p* < 0.01, *** indicates *p* < 0.001 and **** indicates *p* < 0.0001).

Twenty-one days post-boost, mice were intranasally challenged with 10 MLD_50_ of Bel/09 (H1N1) or X-31 (H3N2) virus. mMH3H1F_02TE and mMH3FH1F_02TE immunogens both conferred 100% protection against the Bel/09 H1N1 virus (Figure 7A). mMH3H1F_02TE immunogen conferred 100% while mMH3FH1F_02TE conferred 80% protection against X-31 H3N2 virus (Figure 7B). The extent of weight loss was also negligible for mice immunized with mMH3H1F_02TE and mMH3FH1F_02TE when challenged with Bel/09 (Figure 7C). With X-31 virus challenge, weight loss was higher (mMH3H1F_02TE: 16% and mMH3FH1F_02TE: 20%, respectively) (Figure 7D). This larger weight loss is consistent with the lower homology of the immunogens with the H3 (85%) relative to the H1 (96%) virus. The unvaccinated groups lack protection against challenge with Bel/09 and X-31 viruses. Hence, drastic weight change was observed for these groups (Figure 7C,D) and all animals were dead by day 8 and 9 respectively (Figure 7A,B).

## 4. Discussion

In the present study, we report the design of two HA ectofusion immunogens, mMH3H1F_02TE and mMH3FH1F_02TE, and show that both can be expressed as soluble trimeric proteins. The mMH3H1F_02TE design involves a single foldon trimerization motif at the C-terminus of H1 ectodomain, while the mMH3FH1F_02TE design consists of two foldons, one at the C-terminus of H3 ectodomain and the other at the C-terminus of H1 hemagglutinin, respectively. The recombinant H3-H1 hemagglutinin ectofusion immunogens were correctly folded and as thermostable as individual HA ectodomains. Interestingly, the mMH3H1F_02TE and mMH3FH1F_02TE ectofusion immunogens were equivalent to individual H1 and H3 ectodomains mixed in equimolar amounts (mMH1_02TE + mMH3_02TE) in terms of immunogenicity and protective efficacy, although in the latter case, the immunogen concentration was double of that in the ectofusion groups. Both ectofusion immunogens elicited H1 and H3 specific humoral immune responses in mice and protected against Bel/09 (H1N1) and X-31 (H3N2) virus challenge. We evaluated the immunogenicity of the designed ectofusion immunogens using hemagglutination inhibition (HI) and virus microneutralization (MN) assays. The hemagglutination inhibition assay detects HA-specific antibodies that inhibit agglutination and are a known correlate of protection [36].

**Figure 8:**
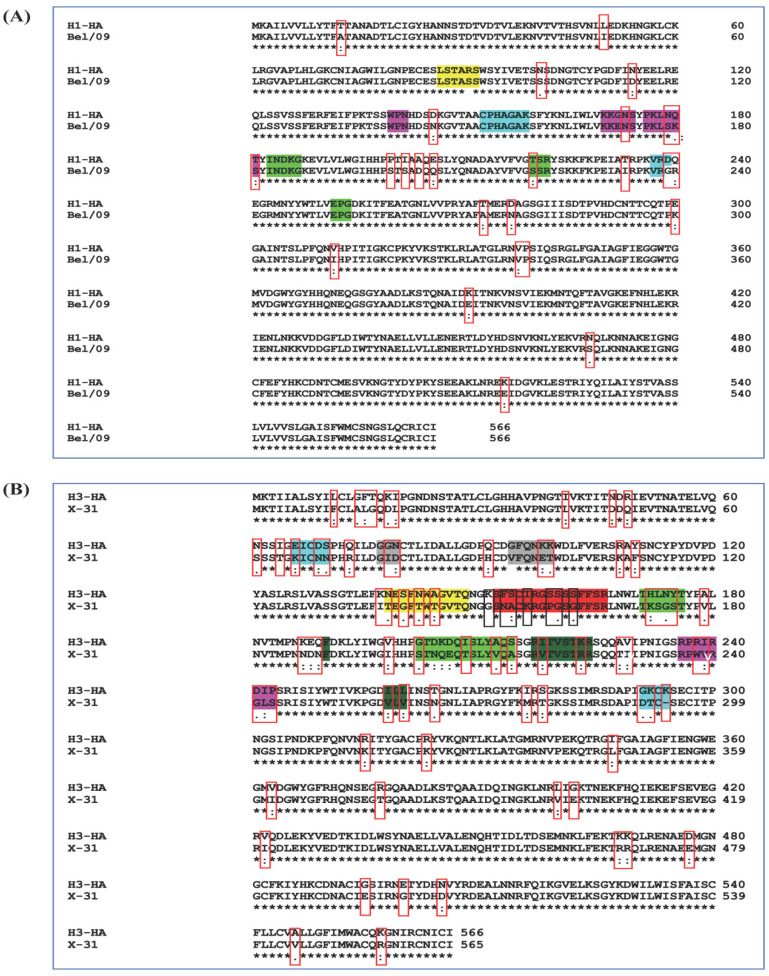
Hemagglutinin sequence alignment using Clustal Omega (1.2.4). (**A**) H1-HA sequence used in ectofusion immunogen design and in challenge virus (Bel/09) were aligned. Antigenic variations are boxed (red color) and antigenic sites are highlighted with different colors (Sa: magenta, Sb: yellow, Ca1: green, Ca2: cyan). (**B**) H3-HA sequence used in ectofusion immunogen design, and in challenge virus (X-31) were aligned. Antigenic variations are boxed (red color) and antigenic sites are highlighted with different colors (Site A: red, Site B: green, Site C: cyan, Site D: dark green, Site E: gray).

The HI titers were high against both H1 viruses (immunogen-matched virus and challenge virus). However, HI titers against H3 were low for both H3 viruses. It is known that the HI assay works less well for recent human H3N2 than for H1N1 viruses [37,38]. The microneutralization assay relies on detecting antibodies that inhibit infection through any mechanism, not just blockage of receptor binding [38]. The microneutralization titers were also high against both challenge-matched H1 virus and homologous H1 virus, and low against challenged-matched and homologous H3 virus. Sequence alignment of H1 and H3 HA from the immunogens with those from challenge virus (Bel/09 and X-31) HA sequences show more antigenic differences in H3 than H1 (Figure 8A,B). In H1, the antigenic variations are largely outside the major antigenic sites, except for the Sa site (antibody recognizing site defined by residues 128–129, 156–160, 162–167), while in H3, variations are present in all antigenic sites A, B, C, D and other regions of the immunogens. This explains the low HI and MN titers and higher morbidity against H3 challenge virus as compared to H1. Surprisingly, even for homologous H3 virus, neutralization titers were low for all sera; possibly the higher glycosylation on H3 HA inhibits elicitation of neutralizing antibodies. In immunized human vaccines, titers against H3 are also lower than against H1 viruses [39,40].

There was complete protection in all groups except for 80% protection observed in the mMH3FH1F_02TE group against H3 X-31 challenge. Higher morbidity was observed against the X-31 challenge relative to the Bel/09 challenge, consistent with the lower homology of immunogen with H3 virus (85%) than H1 virus (96%) and the presence of several mutations in known neutralizing epitopes in the H3 challenge virus. As a consequence of these mutations, antibodies generated against the antigen will poorly neutralize the heterologous challenge virus, thereby lowering protective immunity.

Overall, our data demonstrate that ectodomain fusion can be tolerated without loss of immunogenicity. This is an important result because it shows proof of principle that such ectofusions are viable immunogens, and this strategy offers a path to including more antigens in the vaccine formulation. However, there is some reduction in antigen yield. Future studies will focus on how other modifications, such as a change in linker length or the use of alternate signal peptides, can be used to address this issue. When only a single HA in the vaccine changes, it is possible that characterizing new HA ectodomain fusion proteins might be more complicated than replacing a single component. The current recombinant Flublok vaccine includes four HA components. If one could successfully make two ectodomain fusion of, for example, H1-H3 influenza A and HY-HV influenza B components, the number of components could be reduced from four to two. It could similarly be applied to additional NA components. It remains an open question as to whether this apparent simplification would come with reduced yield and other issues. The present study is a proof of principle that ectodomain fusion is possible without loss of conformational integrity. Much further work needs to be done to examine if this process is robust to the precise identity of the ectodomains and whether this approach will actually find utility in real world influenza vaccines.

Recombinant ectofusion immunogen designs would potentially be advantageous for vaccine manufacturers since they reduce the number of components that need to be GMP manufactured. However, as with seasonal vaccines, these HA ectofusion immunogens would need to be changed annually to accommodate recommended strain changes in annual influenza vaccine formulations. This is certainly an inconvenience if only a single strain changes in the formulation, and only a single ectofusion is used. However, if a similar approach also works for the two influenza B components and the ectofusion approach is robust to minor changes in individual HA sequences, then this would facilitate incorporation of other protective antigens in the vaccine formulation as discussed below. These HA ectofusion molecules will likely provide only sub-type or strain-specific protection [41]. Given the requirement to provide broad protection, the focus of immunogen design in recent years has been to include conserved epitopes in the hemagglutinin stem, hemagglutinin receptor binding site, and to alternative immunogens, such as neuraminidase (NA), M2 extracellular domain (M2e), and internal proteins (PB1, NP, and M1) [15,42,43,44]. Antibodies against neuraminidase, the other major surface glycoprotein of influenza, are also known to protect against influenza virus infection [45]. In addition to HAI titers, NAI titers have also been identified as a correlate of protection [17].

Recently broadly protective anti-NA antibodies have been identified against influenza A and B viruses [18,19,46]. Antibodies to M2e, the terminal extracellular domain of the M2 protein, are also known to confer broad protection [15,20,21,22,47]. These conserved antigens are promising candidates for vaccine design, but likely cannot be used as stand-alone vaccines [15]. However, these antigens can be used in combination with HA to potentially improve and broaden protection against influenza [43,48]. One barrier to include these antigens is the technical difficulty of adding additional components to current quadrivalent vaccine formulation, since each component needs to be separately manufactured. This difficulty might be overcome with our ectofusion designs. Since such recombinant HA ectofusion designs would reduce the total number of HA components, this would enable incorporation of other components in the vaccine formulation. Such vaccines are expected to have better efficacy than current seasonal vaccines.

## Figures and Tables

**Table 1:** Hemagglutinin ectofusion immunogen binds broadly neutralizing antibodies (bnAbs) with high affinity. Kinetic parameters for conformation-specific bnAbs to HA ectofusion immunogens by SPR. * ND: No dissociation. k_on_ error measurement values are <±0.01 × 10^5^.

Neutralizing Antibody	Kinetic Parameters	Immunogens	
		mMH3H1F_02TE	mMH3FH1F_02TE
MA2077	k_a_ (M^−1^s^−1^)	1.3× 10^5^	2.0 × 10^4^
	k_d_ (s^−1^)	3.0 × 10^−4^ ± 0.1 × 10^−4^	1.8 × 10^−4^ ± 0.1 × 10^−4^
	K_D_ (M)	2.4 × 10^−9^ ± 0.1 × 10^−9^	9.0 × 10^−9^ ± 0.7 × 10^−9^
C05	k_a_ (M^−1^s^−1^)	1.8 × 10^5^	3.4 × 10^4^
	k_d_ (s^−1^)	ND *	ND *
	K_D_ (M)	ND *	ND *
CR9114	k_a_ (M^−1^s^−1^)	1.8 × 10^5^	5.6 × 10^4^
	k_d_ (s^−1^)	6.3 × 10^−5^ ± 0.8 × 10^−5^	9.8 × 10^−5^ ± 0.4 × 10^−5^
	K_D_ (M)	3.4 × 10^−10^ ± 1.2 × 10^−10^	1.7 × 10^−9^ ± 0.7 × 10^−9^

## Data Availability

All the data are included in this article and the supplementary file.

## Supplementary Materials

The following are available online at https://www.mdpi.com/article/10.3390/v13091710/s1, Table S1: ELISA, HI, and MN titers in mice sera, immunized with hemagglutinin ectofusions or ectodomain mixtures.
