# Supplementary material for: Protective Efficacy of Recombinant Influenza Hemagglutinin Ectodomain Fusions
Source: Viruses. 2021 Aug 27;13(9):1710. doi: 10.3390/v13091710 (PMC8473191; doi:10.3390/v13091710)
Supplement: Supplementary file 1 [file viruses-13-01710-s001.zip › Supplementary Table.pdf]

Table S1: ELISA, HI, and MN titers in mice sera, immunized with hemagglutinin ectofusions or ectodomain mixtures

Immunogen	Groups	Mice	Elisa Endpoint Titers		HI Titers						MN Titers	
			mMH1_02 TE Tagless	mMH3_02 TE Tagless	A/Guangdong-Maonan/SWL1536/2019	A/Hong Kong/2671/2019	A/Belgium/145-MA/2009	A/Aichi/2/68	A/Guangdong-Maonan/SWL1536/2019	A/Hong Kong/2671/2019	A/Belgium/145-MA/2009	A/Aichi/2/68
mMH3H1F_02 TE	I	1	409600	102400	160	< 10	160	< 10	640	-	160	20
		2	102400	102400	320	20	640	< 10	1280		320	20
		3	102400	102400	160	20	320	< 10	320		640	10
		4	102400	102400	640	10	640	< 10	320		640	10
		5	102400	409600	640	10	1280	< 10	320		640	10
	II	6	102400	102500	640	40	1280	10	-	160	320	10
		7	102400	25600	320	10	160	< 10		160	320	20
		8	102400	25600	320	20	1280	< 10		80	160	10
		9	102400	102400	160	0	1280	< 10		80	320	20
		10	25600	25600	320	10	320	< 10		80	320	10
	GMT		102400	77612	320	<10	557	<10	485	105	342	13
mMH3FH1F_02 TE	III	1	409600	102400	1280	20	1280	< 10	320	-	160	10
		2	409600	102400	320	40	1280	< 10	640		320	10
		3	102400	102400	640	40	1280	< 10	640		320	10
		4	409600	102400	640	160	1280	< 10	160		640	20
		5	102400	102400	320	40	1280	< 10	320		160	10
	IV	6	102400	25600	640	20	1280	10	-	160	640	20
		7	409600	102400	640	40	320	< 10		40	640	10
		8	102400	102400	320	40	320	10		80	640	10
		9	102400	102400	320	160	1280	< 10		20	1280	20
		10	102400	102400	1280	80	1280	< 10		80	1280	20
	GMT		178289	89145	557	49	940	<10	368	61	485	14
mMH1_02TE + mMH3_02TE	V	1	409600	25600	1280	10	1280	10	640	-	320	20
		2	102400	25600	640	20	1280	< 10	640		320	20
		3	102400	25600	160	< 10	640	10	1280		640	40
		4	102400	25600	640	10	640	< 10	640		160	20
		5	102400	25600	160	10	320	< 10	1280		320	20
	VI	6	409600	25600	1280	10	1280	< 10	-	40	1280	20
		7	102400	102400	640	10	1280	10		80	640	20
		8	102400	25600	640	10	1280	< 10		80	1280	20
		9	102400	6400	320	10	160	10		20	320	20
		10	102400	6400	320	< 10	160	< 10		40	160	10
	GMT		135117	22286	485	11	640	<10	844	46	422	20
